# Dental Fear and Anxiety in Children: Association with Individual and Familiar Factors Using Structural Equational Modelling

**DOI:** 10.3390/ijerph23050592

**Published:** 2026-04-30

**Authors:** Ana Clara F. Paiva, Jéssica M. Bittencourt, Daniela Rabelo-Costa, Jennifer E. Gallagher, Saul M. Paiva, Cristiane B. Bendo

**Affiliations:** 1 Department of Pediatric Dentistry, Faculty of Dentistry, Federal University of Minas Gerais (UFMG), 6627 Av. Antônio Carlos, Belo Horizonte 312070-901, MG, Brazil; acfp2014@ufmg.br (A.C.F.P.); danielarabeloodonto@ufmg.br (D.R.-C.); smpaiva@ufmg.br (S.M.P.); crysbendo@ufmg.br (C.B.B.); 2 Dental Public Health, Centre for Host Microbiome Interactions, Faculty of Dentistry, Oral & Craniofacial Sciences, King’s College London, Bessemer Rd., London SE5 9RS, UK; jenny.gallagher@kcl.ac.uk

**Keywords:** dental anxiety, dental fear, child, caregiver, structural equation modelling

## Abstract

**Highlights:**

**Public health relevance—How does this work relate to a public health issue?**

- This study addresses an important gap in public health research by including a substantial sample of children capable of providing self-reported data, which is uncommon for this age group.
- Additionally, a large proportion of participants had never accessed dental care, highlighting barriers to oral health services and the vulnerability of this population.

**Public health significance—Why is this work of significance to public health?**

- Dental fear and anxiety are highly prevalent across populations and are influenced by multiple factors. This study underscores the importance of adopting an individualised approach when assessing children, particularly by considering psychosocial determinants such as maternal depression.
- Furthermore, it focuses on a population with unmet oral health needs, reinforcing its relevance to public health interventions.

**Public health implications—What are the key implications or messages for practitioners, policy makers and/or researchers in public health?**

- The findings provide important evidence to inform policymakers, particularly in the Municipality of Carmópolis de Minas and similar settings, about the need to prioritise children’s oral health. The high prevalence of dental caries and the dependence on public health services highlight the urgent need for targeted preventive and care strategies.
- For researchers, this study offers an innovative methodological contribution by incorporating children’s self-reports of dental fear and anxiety along with structural equation modelling. This approach enables the simultaneous assessment of multiple associated factors, providing a more comprehensive understanding of the determinants of dental fear and anxiety.

**Abstract:**

Dental fear and anxiety (DFA) are common among children, and their occurrence needs to be assessed in population-based studies. This study aimed to evaluate a structural model for the association of DFA in children with individual and family-related factors. A representative cross-sectional study was conducted in Carmópolis de Minas, Brazil, with 272 children aged 4–6 years. Children’s DFA was self-reported using the Children’s Fear Survey Scale—Dental Subscale, and caregivers' DFA using the Dental Fear Survey. Caregivers provided sociodemographic information on the family and children’s health. Two calibrated dentists examined the children for dental caries using the DMFT index. Structural equation modelling was performed. Dental caries showed a direct association with greater DFA in children (β = 0.145; *p* = 0.036). Toothache showed an indirect association with children’s DFA through the variable “dental visit”, as children with toothache had visited the dentist more recently (β = 0.401; *p* < 0.001), and a more recent dental visit was associated with DFA (β = −0.158; *p* = 0.037). Maternal depression was associated with greater caregiver DFA (β = 0.124; *p* = 0.006), but no association was found between children’s DFA and caregiver’s DFA (*p* > 0.05). Children with caries-related toothache who visited the dentist within six months before data collection—and more recently—had higher DFA.

## 1. Introduction

Dental fear and anxiety (DFA) is a recognised barrier to dental care and globally prevalent among children and adolescents, with an estimated worldwide average prevalence of 23.9% (95% CI: 20.4–27.3) [1]. Studies in Brazil report DFA prevalence in children ranging from 15% to 39% [2,3,4,5]. Differences in prevalence rates may be influenced by cultural and contextual factors, as well as variations in methodological approaches used across studies.

Dental caries is one of the most prevalent dental diseases in children globally and in Brazil [6,7]. Regular dental visits are recommended to support the prevention and early detection of disease. DFA is characterised as strong negative emotions associated with the dental environment. These feelings may arise from direct experiences involving real threats, such as the use of drills or anaesthetics, or from anticipatory thoughts about potential negative events during a dental appointment [8]. People who avoid dental treatment because of fear tend to present worse oral health, require more invasive treatment, and likely experience increased apprehension in the next appointment [9,10,11]. The frequent occurrence of DFA affects dental visits, as fearful patients tend to attend fewer appointments, limiting opportunities for preventive care [12]. Therefore, it may negatively impact individuals’ oral health and their oral health-related quality of life [12,13].

Fear acquisition in childhood occurs through three primary pathways: direct conditioning, vicarious learning, and informational transmission [14]. As proposed by Rachman (1977), these pathways are related to children’s experiences, modelling, and learning [14]. The experiential pathway involves learning through personal experiences. Children who experienced painful or other aversive situations may carry these feelings into future dental visits, resulting in elevated dental fear and anxiety [15,16]. Dental fear and anxiety has also been associated with oral health, with higher prevalence among children with caries experience compared to their caries-free counterparts [1,17]. The modelling pathways refer to the acquisition of fear through observation, particularly of caregivers. Children whose caregivers have higher levels of fear and anxiety tend to show similar patterns [18,19]. Finally, the learning pathway involves the indirect acquisition of dental fear and anxiety through external information sources, such as media, cartoons, or others’ negative accounts of dental care shared [14,20].

Various studies have explored these mechanisms in the development of DFA, as well as the influence of background and endogenous factors [3,21,22,23]. Evidence supporting direct conditioning as a strong catalyst for DFA is robust [10,15,17,24]. Children with negative experiences tend to present higher DFA during new appointments [15,16]. Moreover, children with dental caries experience and those whose parents have DFA show higher DFA [1,3,17]. Other factors, including personality traits, family factors such as lower socioeconomic level, and cultural values, likely contribute to the maintenance of DFA [24,25].

Although many questions about DFA have been clarified, it remains important to explore certain aspects regarding the onset of DFA. One key area to investigate is trait anxiety, measured outside the clinical setting, which evaluates a more permanent characteristic of DFA rather than a situation caused by specific stimuli. Early detection and treatment can prevent children from carrying DFA into adulthood [26]. This approach allows for the analysis of different dimensions of DFA. Clinicians can discuss intervention strategies tailored to each child’s profile and select the most appropriate technique to reduce DFA throughout childhood. This strategy will help combat avoidance and negative impacts on the oral health of fearful children. Further investigation is needed into how family and background factors relate to this phenomenon, especially in children aged 4 to 6, as the existing literature remains scarce [25]. The findings emphasise the importance of further research on parent–child relationships from early infancy.

Previous studies relied on classical statistical approaches, which limited the exploration of complex dependency relationships among variables and the identification of direct and indirect pathways associated with children’s DFA [5,16]. These aspects need to be investigated using more advanced statistical methods, such as structural equation modelling, which can analyse multiple dependency relationships simultaneously and more reliably, as measurement error is taken into account [27].

The aim of this study was to investigate children’s self-reported DFA and its association with endogenous and exogenous factors related to both children and their caregivers through structural equation modelling. The first hypothesis was that children’s individual characteristics and external factors, such as prior dental experiences, oral health issues, and dental visits, are directly associated with children’s DFA. The second hypothesis was that the dental fear and anxiety experienced by caregivers are indirectly associated with the DFA experienced by children.

## 2. Materials and Methods

The present study conforms to the Strengthening the Reporting of Observational Studies in Epidemiology (STROBE statement) [28].

### 2.1. Ethical Requirements

This study was approved by the Human Research Ethics Committee of the Federal University of Minas Gerais (certificate number: 31334720.1.0000.5149) in accordance with Resolution 466/12 of the National Board of Health. Parents or caregivers signed an informed consent form, and their children signed the informed assent form. The municipal education department and all schools provided their formal agreement.

### 2.2. Study Design, Sample Selection, and Eligibility Criteria

An observational cross-sectional study was conducted in the municipality of Carmópolis de Minas, Minas Gerais, Brazil, from March to September 2023. The city is located 120 kilometres from Belo Horizonte, the state capital.

Carmópolis de Minas has approximately 18,000 inhabitants, and there were 619 children enrolled in the first and second years of preschool education and the first year of primary school across seven preschools and schools. Children aged 4 to 6 years from private and public schools, enrolled in these grades, were selected. To ensure the representativeness of the study, every caregiver of the 619 children was contacted to participate. Children with cognitive impairments reported by teachers or family were excluded from the sample due to their inability to answer the questionnaires.

The sample size was calculated with a 5% acceptable margin of error, a 95% confidence level, and an assumed prevalence of DFA of 36.5% based on a previous study [1]. Simple random sampling was performed. The minimum sample size was 225 preschoolers, increased by 20% (n = 281) to compensate for possible losses.

### 2.3. Calibration

The calibration of the two examiners for dental caries experience was performed in both theoretical and clinical steps, coordinated by a specialist considered the gold standard. For the theoretical step, the DMFT (decayed, missing, filled teeth) index from the World Health Organization (WHO) was studied using official documents presented by the gold standard specialist, and photos were used for initial calibration. During the clinical calibration phase, five children were examined using the DMFT index by three examiners: the gold standard specialist and two examiners who were master’s students. After 15 days, the same children were examined again to calculate intra-rater agreement. High concordance is observed when the Kappa value is greater than 0.81 [29]. Examiner #1 achieved a Kappa value of 0.933 when compared with the gold standard examiner, while Examiner #2 achieved a Kappa value of 0.892. The inter-examiner Kappa coefficient between Examiners #1 and #2 was 0.828. After 15 days, the intra-examiner Kappa values were 0.959 for Examiner #1 and 0.809 for Examiner #2. All Kappa values were considered satisfactory.

### 2.4. Pilot Study

The pilot study involved 28 pairs of children and caregivers, who were not included in the final sample. This step was intended to verify the methods and data collection instruments, which were found to be adequate.

### 2.5. Main Fieldwork: Data Collection and Variables

First, parents and caregivers were invited to participate in the study through a school mailing that included a letter outlining the study details, the consent form, and questionnaires. Children whose caregivers agreed to participate and completed the questionnaires were asked for their assent, then completed their own questionnaire and underwent a dental examination in a designated room at each school.

#### 2.5.1. Latent Variable

To measure children’s self-reported DFA, the Children’s Fear Survey Scale—Dental Subscale (CFSS-DS) was used. The CFSS-DS assesses aspects of dental care, including clinical and psychological characteristics. The 15 items are rated on a five-point scale from one (not afraid) to five (very afraid). The total score ranges from 15 to 75, with higher scores indicating greater DFA [30]. This study used the Brazilian adapted and validated version of the CFSS-DS [31]. A facial image scale is presented alongside the five-point scale to help younger children express their feelings [31]. The children answered the questionnaire during interviews conducted by the examiners.

#### 2.5.2. Observable Variables

Caregivers’ dental fear was measured as an observable variable using the question “How fearful are you of having dental work done?”. This question reflects an overall self-evaluation of how afraid the adult is of dentists, rated from 1 (not afraid) to 5 (very afraid).

Sociodemographic data was collected using a questionnaire completed by the caregivers, addressing characteristics of both the caregivers and the children. Children were categorised as female or male, and age was recorded in years.

A child’s last dental visit was categorised into five time periods: never, more than two years ago, between one and two years ago, between six months and one year ago, and less than six months ago. Maternal education was assessed by school grades, ranging from no formal education to completion of higher education. Maternal depression and caregivers’ negative dental experience were categorised as yes or no. This information was obtained through caregiver self-report in response to questions about depression and dental experience in the sociodemographic instrument.

The DMFT index (WHO) was dichotomised into children with dental caries experience and those without. Dental examinations were conducted by two trained examiners. Clinical examinations were carried out using a clinical mirror (Golgran Indústria e Comércio de Instrumental Odontológico Ltda., São Paulo-SP, Brazil), WHO probe (Golgran Indústria e Comércio de Instrumental Odontológico Ltda., São Paulo-SP, Brazil), artificial light (Petzl Zoom, Clifton Park, NY, USA), and individual protective equipment. Each child was called out of their classroom and guided to a separate room in the school, where they were seated in a school chair facing the examiner, who was also seated in a school chair. Children identified as having a dental need received a note advising their guardians to seek care at a basic health unit.

#### 2.5.3. Statistical Analysis

Descriptive statistics were performed using IBM SPSS Statistics (IBM Corp., released 2019, IBM SPSS Statistics for Windows, version 26.0, Armonk, NY, USA). Confirmatory Factor Analysis (CFA) and Structural Equation Modelling (SEM) were conducted with Mplus (version 8.6, Muthen & Muthen, Los Angeles, CA, USA).

A measurement model for the latent variable was developed using CFA to evaluate the plausibility of the CFSS-DS structure.

SEM was conducted to identify simultaneous relationships among factors associated with children’s DFA, based on a conceptual model developed by the authors. Furthermore, SEM accounts for measurement error, unlike traditional regression methods, thereby providing greater reliability to the results obtained [27].

**Figure 1:**
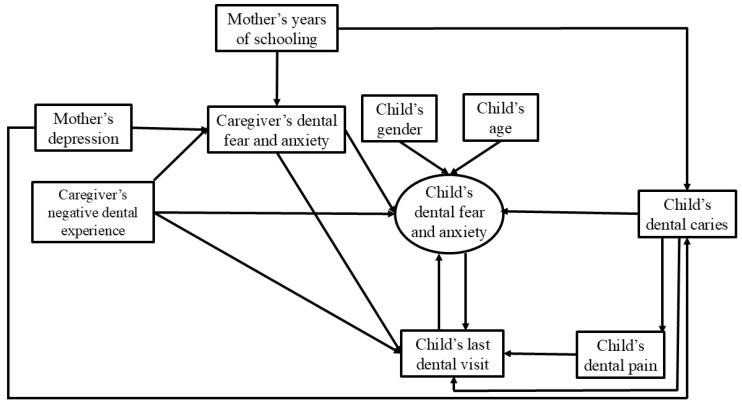
Conceptual model elaborated by the authors.

The conceptual model was developed from theoretical models [14,24] and consolidated by previous studies (Figure 1). The associations between children’s age, sex, dental caries, dental pain, and last dental visit with DFA were supported by previous studies and reinforced the pathways proposed by Rachman, such as direct condition [13,15,16,18,19,21,24]. Maternal aspects were also investigated based on the modelling pathways proposed by Rachman and supported by other studies [1,3,14,17].

CFA and SEM were conducted using the weighted least squares mean and variance-adjusted (WLSMV) estimation method, which is appropriate for categorical data [32]. The following goodness-of-fit indices were used to evaluate the measurement and structural models: Comparative Fit Index (CFI), Tucker–Lewis Index (TLI), and Root Mean Square Error of Approximation (RMSEA). CFI and TLI values should be > 0.90, while RMSEA values should be < 0.08 [33]. Effect sizes in the structural equation model were assessed using standardised coefficients (β). Values around 0.10 were considered small, approximately 0.30 moderate, and ≥0.50 large [33]. Additionally, clinical significance was considered when the magnitude of the association was significant for clinical decision-making in paediatric dentistry. An association was considered clinically relevant when (a) the magnitude of the standardised coefficient indicated a meaningful difference for children and parents/caregivers; (b) the association involved outcomes with practical implications, such as dental fear, pain, or dental care utilisation; and (c) the association was statistically significant [34,35].

## 3. Results

A total of 272 children self-reported their DFA. Girls comprised 57.0% of the sample. Nearly half of the children (46.3%) had at least one tooth affected by dental caries. Children who had never visited a dentist accounted for 32.7% of the sample, while 35.3% had visited a dentist in the six months prior to the study. Most children had never experienced dental pain (93.0%). Most caregivers were classified as having no fear (39.0%) or little dental fear (32.0%) (Table 1).

**Figure 2:**
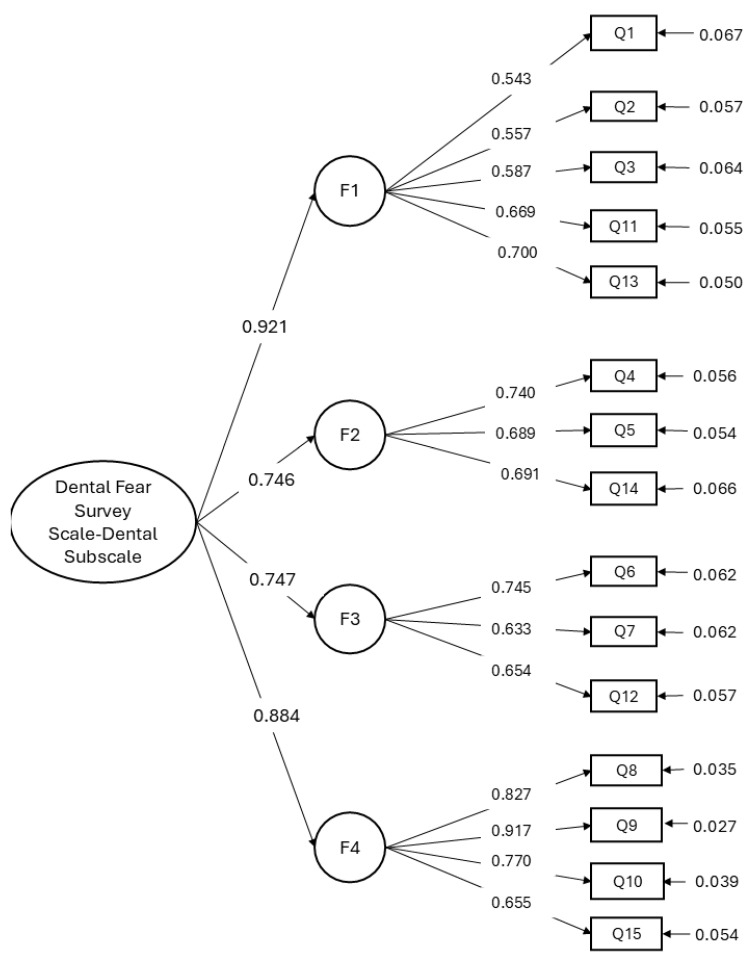
Multidimensional structure of the CFSS-DS.

The multidimensional structure of the CFSS-DS instrument showed a good fit to the data, with goodness-of-fit indices supporting the model: CFI = 0.956; TLI = 0.947; RMSEA = 0.061 (0.047–0.074) (Figure 2).

**Figure 3:**
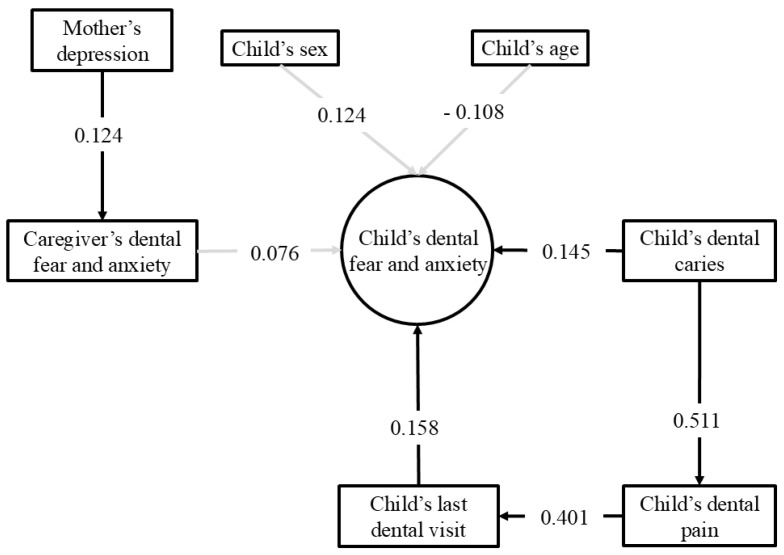
Structural equation modelling with standardised coefficients. Pathways in bold: *p <* 0.05.

Table 2 shows the initial and final structural models. The models demonstrated an adequate overall fit, suggesting their plausibility. According to the final SEM (Figure 3), children with dental caries experience had higher self-reported DFA (β = 0.145; *p* = 0.036). Dental pain was indirectly associated with children’s self-reported DFA through the variable ‘last visit to the dentist’, with children experiencing dental pain having visited the dentist more recently (β = 0.401; *p* < 0.001), and a more recent visit to the dentist being associated with DFA (β = −0.158; *p* = 0.037). Maternal depression was associated with mothers’ self-reported DFA (β = 0.124; *p* = 0.006). There was no association between caregivers’ and children’s variables (*p* > 0.05).

Most associations showed small effect sizes. The association between toothache and last dental visit showed a moderate effect size, while the association between tooth decay and toothache showed a large effect size, indicating varying magnitudes of association between the variables in the model.

## 4. Discussion

The results indicated that the structural model in this study adequately fitted the data. Factors associated with DFA included dental caries experience, which had a direct association, while dental pain was indirectly associated through the variable of the last dentist visit. Moreover, caregivers’ self-reported depression was associated with higher DFA in caregivers. Approximately one-third of children aged 6 or younger experience DFA worldwide, and various background theories are proposed based on the associated factors [24]. Since Rachman’s pathways for fear acquisition, studies have tested associations related to DFA and developed reviews to consolidate these findings and illustrate the theoretical backgrounds for DFA [14,15,21,23,24].

Dental caries experience was directly associated with DFA in this sample, confirming that oral health status plays a significant role in DFA. Previous studies have also shown that children with caries experience have a higher prevalence of DFA than caries-free children [1,17,36]. The present results were obtained through children’s self-reports of DFA, using an assessment tool that includes various aspects of DFA, unlike other studies that rely on caregivers’ or dentists’ proxy reports of the child’s DFA, or use a single question about DFA [2,3,5]. The findings of this study reinforce the importance of allowing children to express themselves, with the caregiver’s report serving as support in developing strategies to address the child’s DFA.

Unlike the present study, variables such as sex, age, and parents’ years of study have been found in the literature to be associated with a child’s DFA [1,4,21]. These results may differ due to the age of the sample. Studies reporting that females have higher DFA usually involve participants aged over 7 years or adults [4].

The conditioning pathway occurs when an individual learns through personal experience. Children with negative past dental experiences carry these into new appointments, exhibiting higher DFA [15,16,22]. Our study found that children experiencing dental pain due to caries attended a dental appointment less than six months before our data collection, and those who visited more recently showed higher DFA. The literature confirms that dental pain is associated with increased levels of DFA [2,36].

The present study demonstrated that mothers’ depression was associated with caregivers’ DFA, corroborating results from another study that found an association between depression and anxiety with DFA in adults [37]. The absence of an association between caregivers’ factors and the child’s DFA suggests that DFA in early childhood may be related to direct conditioning rather than modelling; this result is in accordance with recent literature [13,38]. During early infancy, children experience what is called a sensitive period, which represents a broad window of vulnerability to potentially harmful effects. During this period, the brain is highly receptive, and negative stimuli can lead to lasting effects. These lasting effects occur not only due to the high vulnerability of this developmental stage, but also due to the cumulative impact of such experiences [39]. The findings suggest that children may face difficulties during a critical stage of development, and early life experiences can produce long-term effects [39,40]. However, the impact of adverse early experiences can be mitigated through policies and programmes that support parents and caregivers. Children in this study have access to care within the public health system, although some face barriers due to the distance between their homes and the city centre for appointments. Carmópolis de Minas lacks paediatric dentists in the public health system, which may negatively affect children’s experiences. Integrated preventive actions involving primary care, local authorities, and community sectors are essential to promote improved child health and development outcomes [40]. This is also relevant for supporting mothers struggling with mental illness. The association between maternal depression and maternal DFA requires intervention to achieve better health outcomes for both mother and child.

The use of statistical methods that enable simultaneous analysis of various factors is helpful in combining these determinants to make a positive and individualised impact on the treatment of DFA in children. This study highlighted that individual factors, such as dental caries and dental pain, are more closely related to DFA, indicating the need to view the patient as a whole rather than focusing solely on the mouth. Providing patients with the opportunity to explain their DFA, adopting patient-centred care by involving them in decision-making, and understanding their perspectives would likely increase the likelihood of patients following dentists’ recommendations [41]. It is important to highlight that, beyond statistical significance, the associations observed in this study also demonstrated clinical relevance. Moderate to large effect sizes were identified for key pathways, particularly those involving dental pain and dental caries, suggesting significant implications for paediatric dental practice and for the management of children’s DFA.

This study has some limitations and highlights areas for improvement in future research. Firstly, different assessment tools were used for children’s and caregivers’ DFA, which may affect the validity of comparisons between the two groups. Moreover, the use of a single item to assess caregivers’ DFA may not adequately capture the multiple dimensions of this construct. It is strongly recommended that future studies employ validated instruments, such as the Dental Fear Scale [42], or use tools designed for both children and parents based on the same theoretical framework to improve comparability and enable more robust analyses of inter-report agreement. Standardisation would also facilitate the inclusion of data in future meta-analyses on children’s DFA. Similarly, the assessment of maternal depression relied on a self-report of past or present history using a single direct question, which is limited as it does not employ a validated instrument capable of capturing the complexity of this construct.

Another limitation is that, despite comprehensive efforts to reach all caregivers, some attrition occurred due to non-responsiveness. Nonetheless, the sample size achieved exceeded the minimum threshold established by the sample calculation. Moreover, Carmópolis de Minas is a small city in the countryside of Minas Gerais. The behaviours of its inhabitants may differ from those in large cities in Brazil and cities in other countries, so caution is needed when generalising the results.

Further research on DFA using samples outside the clinical setting is needed in other cultures. Moreover, this study underscores the importance of prioritising children’s self-reports in clinical and research settings, allowing for a more accurate and empathetic understanding of their experiences. Subjective assessment methods are practical, quick to administer, and do not require invasive procedures, while maintaining robust accuracy and consistency. They are versatile and suitable for various environments and for children across different age groups [43]. These findings support the practice of individualised, patient-centred dental care that takes into account the child’s background, emotional state, and lived experiences. Addressing the DFA from both clinical and psychological perspectives may contribute to more effective interventions and improved long-term oral health outcomes.

## 5. Conclusions

In conclusion, this study reinforces the multifactorial nature of DFA in early childhood, emphasising the association of clinical factors such as dental caries, dental pain, and dental visits. Dental caries emerged as a direct determinant of DFA, while dental pain demonstrated an indirect effect through recent dental visits. The representativeness of the study, including an assessment of trait anxiety via child self-report in a sample outside the clinical setting, is an important contribution to the paediatric dentistry literature.

## Figures and Tables

**Table 1:** Descriptive statistics of the variables included in the study.

Variables	N (%)
Sex	
Male	117 (43.0)
Female	155 (57.0)
Age	
4	57 (21.0)
5	108 (39.7)
6	107 (39.3)
Dental caries in the child	
Yes	126 (46.3)
No	146 (53.7)
Child’s last dental visit	
Never	89 (32.7)
More than two years	11 (4.0)
Between one and two years	19 (7.0)
Between six months and one year	57 (21.0)
Less than six months	96 (35.3)
Child’s dental pain	
Yes	19 (7.0)
No	253 (93.0)
Respondents *	
Mother	257 (95.5)
Father	8 (3.0)
Others	4 (1.5)
Mother’s dental fear *	
None	106 (39.0)
A little	87 (32.0)
Sometimes	40 (15.1)
Afraid	21 (7.7)
Very much afraid	17 (6.3)
Mother’s schooling	
0 to 8 years	51 (18.7)
Incomplete high school	34 (12.5)
Complete high school	122 (44.9)
Incomplete undergraduate	14 (5.1)
Complete undergraduate	51 (18.8)
Mother’s depression	
Yes	19 (7.0)
No	253 (93.0)
Mother’s negative dental experience *	
Yes	53 (20.9)
No	200 (79.1)

* Missing data is due to incomplete questionnaires.

**Table 2:** Standardised coefficients of structural equation modelling.

Ways	Initial Model Standardised Coefficients	Final Model Standardised Coefficients	Confidence Interval	Standard Error
	(*p*-Value)	(*p*-Value)		
Child’s Dental Fear ON				
Age	−0.110 (0.143)	−0.108 (0.126)	−0.289–0.074	0.07
Sex	0.137 (0.053)	0.124 (0.066)	−0.050–0.298	0.068
Child’s last dental visit	−0.332 (0.250)	−0.158 (0.037)	−0.353–0.037	0.076
Child’s dental caries	0.224 (0.061)	0.145 (0.036)	−0.033–0.324	0.069
Mother’s dental fear	0.081 (0.195)	0.076 (0.215)	−0.082–0.233	0.061
Mother’s negative dental experience	0.034 (0.705)	-	-	-
Child’s last dental visit ON				
Child’s dental fear	0.184 (0.534)	-	-	-
Child’s dental caries	−0.027 (0.863)	-	-	-
Child’s dental pain	0.424 (0.004)	0.401 (<0.001)	0.171–0.631	0.089
Mother’s negative dental experience	−0.041 (0.563)	-	-	-
Mother’s DFA	−0.023 (0.737)	-	-	-
Child’s dental caries ON				
Mother’s schooling	0.063 (0.490)	-	-	-
Mother’s depression	0.072 (0.350)	-	-	-
Mother’s dental fear ON				
Mother’s negative dental experience	−0.022 (0.931)	-	-	-
Mother’s schooling	−0.038 (0.640)	-	-	-
Mother’s depression	0.123 (0.006)	0.124 (0.006)	0.008–0.239	0.045
Child’s dental pain ON				
Child’s dental caries	0.628 (<0.001)	0.511 (<0.001)	0.0341–0.681	0.066
General Fit Indices				
CFI	0.935	0.958	-	-
TLI	0.926	0.953	-	-
RMSEA (IC95%)	0.043 (0.033–0.052)	0.038 (0.026–0.048)	-	-

CFI: comparative fit index; TLI: Tucker–Lewis index; RMSEA: root mean square error of approximation.

## Data Availability

The qualitative datasets generated and/or analysed during the current study are not publicly available due to participant privacy and confidentiality, but are available from the corresponding author on reasonable request.

## Abbreviations

The following abbreviations are used in this manuscript:

CFA	Confirmatory factorial analysis
CFI	Comparative fix index
CFSS-DS	Children Fear Survey Scale–Dental Subscale
CI	Confidence interval
DFA	Dental fear and anxiety
DMFT	Decayed, missing, filled teeth
RMSEA	Root mean square error of approximation
SEM	Structural equation modelling
TLI	Tucker–Lewis index
WHO	World Health Organization
WLSMV	Weighted least squares mean and variance adjusted
